# The effects of transcutaneous electrical nerve stimulation during the first stage of labor: a randomized controlled trial

**DOI:** 10.1186/s12884-021-03625-8

**Published:** 2021-02-24

**Authors:** Anne Njogu, Si Qin, Yujie Chen, Lizhen Hu, Yang Luo

**Affiliations:** 1 Xiangya School of Nursing, Central South University, Changsha, China; 2 Department of Nursing, Hunan Provincial People’s Hospital, the First Affiliated Hospital of Hunan Normal University, Changsha, China

**Keywords:** Transcutaneous electrical nerve stimulation, Labor pain, Labor duration, Pain relievers, Non-pharmacological therapy

## Abstract

**Background:**

Labor pain during childbirth can have devastating effects on the progress of labor, mother, and fetus. Consequently, the management of labor pain is crucial for the well-being of the mother and fetus. Transcutaneous electrical nerve stimulation (TENS) is a non -pharmacological analgesic technique. It uses a low-voltage electrical current to activate descending inhibitory systems in the central nervous system to relieve pain. This study aimed to determine the effects of TENS therapy in the first stage of labor.

**Methods:**

In this single-blind randomized controlled trial, we screened low-risk pregnant women who anticipated spontaneous vaginal delivery. Women were assigned (1:1) to either the experimental group (received TENS therapy in the first stage of labor) or the control group (received routine obstetric care). The women, midwives, and researchers working in the gynecology and obstetric department were aware of the treatment group, but statisticians analysis the data were blinded. The primary outcome was labor pain intensity, assessed by visual analog scale (VAS) immediately after the randomization, at 30, 60, and 120 min after TENS therapy, and 2–24 h post-delivery. We used SPSS 21.0 software in data analysis. An independent sample t-test compared the mean VAS scores and labor duration between groups. A Chi-square test was employed to compare categorical variables between the groups. A significant level of ≤0.05 was statistically significant.

**Results:**

A total of 326 pregnant women were eligible: experimental group (*n* = 161) and control group (*n* = 165). The experimental group had statistically significantly lower mean VAS scores at a different time (30, 60, and 120 min post-intervention and 2–24 h post-delivery) than the control group (*p* < 0.001). The experimental group demonstrated a statistically significant shorter duration of the active labor phase than the control group (*p* < 0.001).

**Conclusion:**

This study indicates that TENS can be used as a non-pharmacological therapy to reduce pain and shorten the active labor phase.

**Trial registration:**

ISRCTN registry, ISRCTN23857995. Registered on 11/12/2020, ‘retrospectively registered.

## Background

According to the International Association for the Study of Pain (IASP), pain is an unpleasant sensory, subjective, and emotional experience associated with actual or potential tissue damage [1]. Like other pain types, labor pain consists of four main processes: transduction, transmission, central representation, and modulation. However, unlike acute and chronic pain experiences, it is associated with a meaningful life experience of bringing forth a life [1, 2]. Labor pain has two elements: visceral and somatic. Visceral pain occurs during the early first stage and the second stage. Nociceptive stimuli from uterine contractions and cervical dilatation are transmitted to the posterior nerve root ganglia at T10 through L1 [3]. Like other visceral pain types, labor pain refers to the abdominal wall, lumbosacral region, iliac crests, gluteal areas, and thighs. Somatic pain occurs during the transitional and the second stage [2, 4]. Painful impulses result from stretching, distension, ischemia, and injury of the pelvic floor, cervix, vagina, and perineum. These stimuli are conducted via the pudendal nerve through the anterior rami of S2 to S4. This pain is sharp and usually well localized [5].

Childbirth pain is universally known as one of the most intense and painful experiences a woman will ever undergo [4]. Numerous factors may influence a woman’s perception of pain in labor, making each experience unique—for example, previous pain experiences, culture, and care provided to mothers during childbirth [2]. Fear of childbirth pain is a common reason pregnant women request an elective cesarean section [6, 7]. Negative physiological consequences of labor pain can have potential effects on the mother, the fetus, and the labor process [8]. These possible effects may include: increased oxygen consumption, maternal nausea, fatigue, respiratory alkalosis, and increased catecholamines production, associated with decreased uterine blood flow, poor uterine contraction, decreased cardiac output and increased blood pressure [8, 9]. Pain relief during labor is essential to reduce its physiological consequences [10].

Neuraxial analgesia is the most effective method in labor pain management but is associated with maternal hypotension, neonatal respiratory depression, and the reduction of neonatal suckling reflexes [11–13]. In contrast, many non-pharmacological therapies appear to be safe, reduce pain intensity, delay pharmacological analgesics, and increase maternal satisfaction [14, 15].

The Transcutaneous Electrical Nerve Stimulation (TENS) is a non-pharmacological and low-frequency electrotherapy technique. The TENS precise mechanism is still unknown, but Melzack and Wall, in 1965, proposed the gate control theory. It was first introduced in obstetrics in 1970 [16–20]. Despite the widespread use of TENS and its potential benefits for the relief of labor pain, evidence from the systematic reviews was inconsistent in demonstrating any significant advantage of this method. Its overall effect in minimizing pain was weak [21–24]. Therefore, we aimed to evaluate TENS therapy’s effects on labor pain intensity and the duration of the active phase of labor.

## Methods

### Study design and participants

This study was a single-blind randomized controlled trial conducted at the Hunan Provincial People’s Hospital (The First Affiliated Hospital of Hunan Normal University) Changsha, Hunan province (China) from March 2017 to June 2017. It is a 3000-bed capacity hospital, and approximately 900 normal deliveries are conducted at the obstetric department annually. The study included pregnant women volunteers aged 18 years and above with the following characteristics: (1) at term (37–42 weeks gestation age), (2) primipara and multipara with no complications during the antenatal period, (3) established active stage of labor, and (4) a single viable fetus in cephalic presentation. We excluded pregnant women with (1) preterm labor, (2) malpresentation, (3) cephalopelvic disproportion, (4) precipitated labor, (5) previous history of cesarean section, (6) antepartum hemorrhage, (7) any medical complications, (8) known fetal abnormalities, (9) multiple gestations, (10) women in the advanced stage of labor, (11) psychiatric disorders, (12) previous history of using TENS, and (13) skin lesions on the electrodes’ application sites.

The researchers conducted this trial following the Declaration of Helsinki and the CONSORT guidelines. It was approved by the Hunan Provincial People’s Hospital (The First Affiliated Hospital of Hunan Normal University) Human Ethics Committee. The principal researcher (LH) obtained the women’s informed consent after explaining the purpose, function, potential advantages, and risks associated with the study. Women were permitted to withdraw from the course at any point in time without affecting their obstetric care. Researchers stated skin redness at the electrode sites was the only possible side effect, and these symptoms would usually disappear spontaneously within a few days.

### Randomization and masking

A researcher (blinded for the group) randomly assigned participants (at an a1:1 ratio) to the experimental group (received TENS therapy in the first stage of labor) or the control group (received routine obstetric care) using a simple technique based on the computer-generated list. Statisticians (AN and SQ) who performed the analysis were blinded from the treatment group. However, the women, midwives, and researchers working in the obstetrics and gynecology department could not be masked.

### Procedures

A midwife and a researcher initiated TENS therapy at the beginning of active labor (4-cm cervical dilatation) until the second labor stage.

**Fig. 1:**
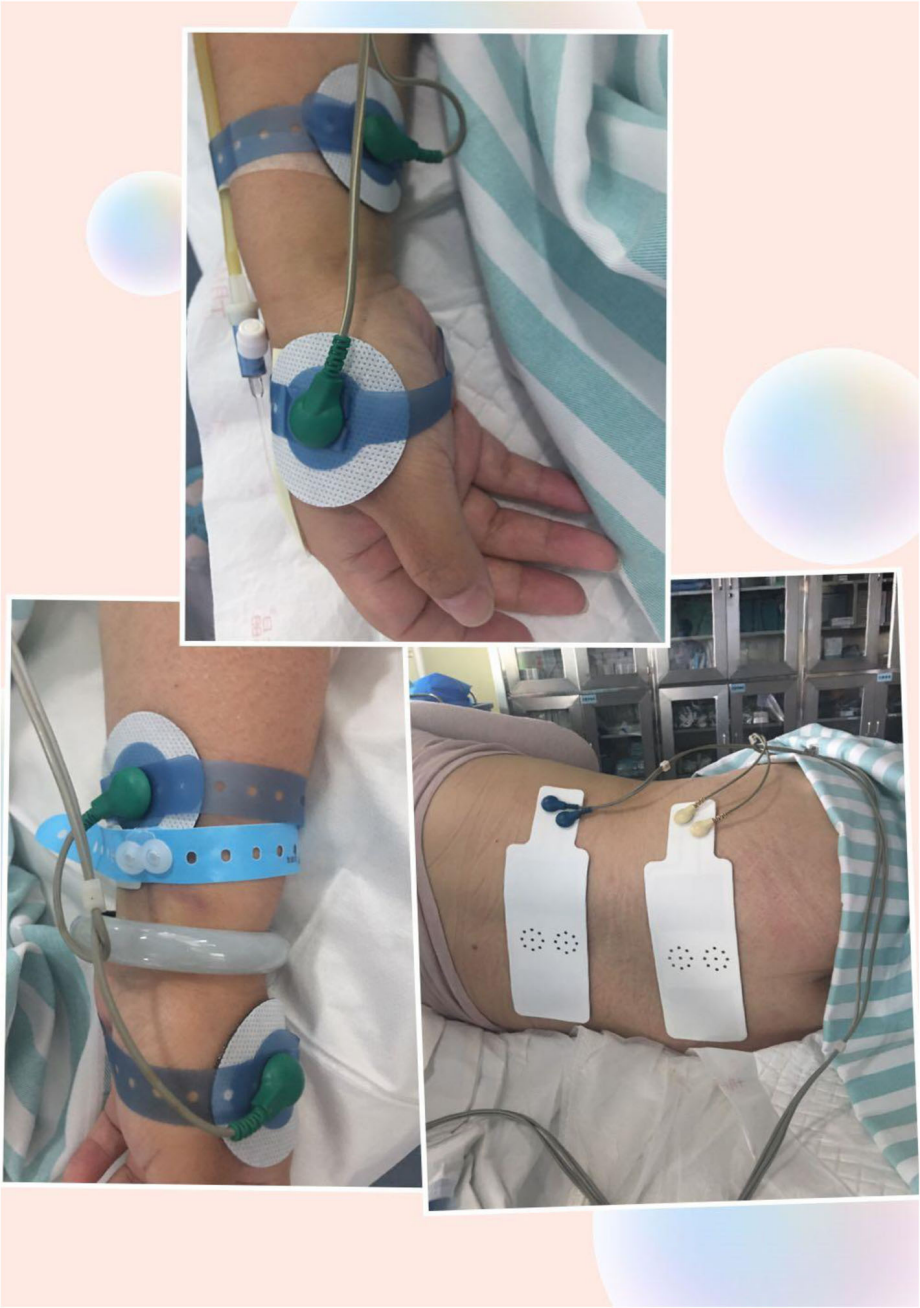
Electrode placement

The researcher only recorded the pain scores immediately after the randomization, at 30, 60, and 120 min after TENS therapy and 2–24 h post-delivery. The intervention used the following procedures: 1) a midwife and a researcher entered participants’ demographic and obstetrics information into a TENS unit system before the intervention; 2) the midwife applied two pairs of electrodes on both arms at hegu points (LI4, the midpoint between first and second carpal bones, first web space dorsal side) and neiguan points (PC6, 4 cm above the medial transverse line in wrist); 3), the midwife placed two electrodes over the participants’ paravertebral regions at the T10–L1 and S2–S4 levels (Fig. 1); 4), two transducers (probes) were placed on the abdomen to monitor fetal heart rate and uterine contractions; 5) finally, the midwife activated the labor analgesia icon. This study used an SRL998A Bio-feed TENS System (Sunray Medical Apparatus CO. LTD. Guangzhou, China). It produced a peak current of 15 mA and a peak open-circuit voltage of 300 V. According to the woman’s maximum tolerance, the midwife adjusted the frequency and intensity of analgesia, characterized by buzzing or pricking sensation without muscle contraction. All pregnant women received standard obstetric care according to the Chinese clinical practice guidelines. Women were encouraged to choose their most comfortable position. One person was allowed to accompany the pregnant woman during labor and delivery.

### Outcomes

The primary outcome was the change in pain severity at the end of the intervention period. We used the visual analog scale (VAS) chart to measure labor pain, where pain scores ranged from 0 (no problem) to 10 cm (worst imaginable pain). The secondary outcomes indicators were obstetric and neonatal findings. Obstetric outcomes included cervical dilatation, oxytocin, duration of the first, second, and third stage of labor, postpartum hemorrhage, pain measurement 2–24 h post-delivery, and any adverse event. At the same time, neonatal outcomes included weight and Apgar scores. Apgar score is a universal rapid and convenient method for reporting the newborn’s status immediately after birth and response to resuscitation if needed. Health care providers perform it at first and fifth minutes and give scores based on muscle tone, heart rate, grimace, appearance, and respiration. Scores of 7–10 are average, 4–6 scores are intermediate, and 0–3 scores are low.

### Sample size calculation

We based our sample size calculation on the primary outcome (pain intensity) and a previous study’s statistical indices [25]. We assumed the mean difference of the VAS score between both groups was 1.2, and the standard deviation was 3. The ratio of the experimental group size to the control group size was 1:1. Therefore, we required 286 women to detect the actual difference with the power of 90% and alpha of 0.05 (two-sided). We used the following formula to calculate the sample size:

$$ \mathrm{N}=\kern0.5em {\left[\frac{\sigma \left({\mu}_a+{\mu}_{\beta}\right)}{\Delta}\right]}^2\kern0.5em \left(\frac{1}{k}+\frac{1}{1-k}\right) $$

N: Sample size; *σ*: Standard deviation; *μ*_*α*_, *μ*_*β*_: Both are boundary values of the standard normal distribution; k, 1-k: the ratios of two groups; Δ: Mean difference.

### Statistical analysis

We used SPSS 21.0 software for statistical analyses. Continuous variables were described by listing the mean and standard deviation, while the categorical variables were presented by listing the case number and percentage. To compare continuous variables between groups, an independent sample t-test, while the chi-square test was employed to compare categorical variables between the groups. *P*-value ≤0.05 was considered statistically significant.

## Results

**Fig. 2:**
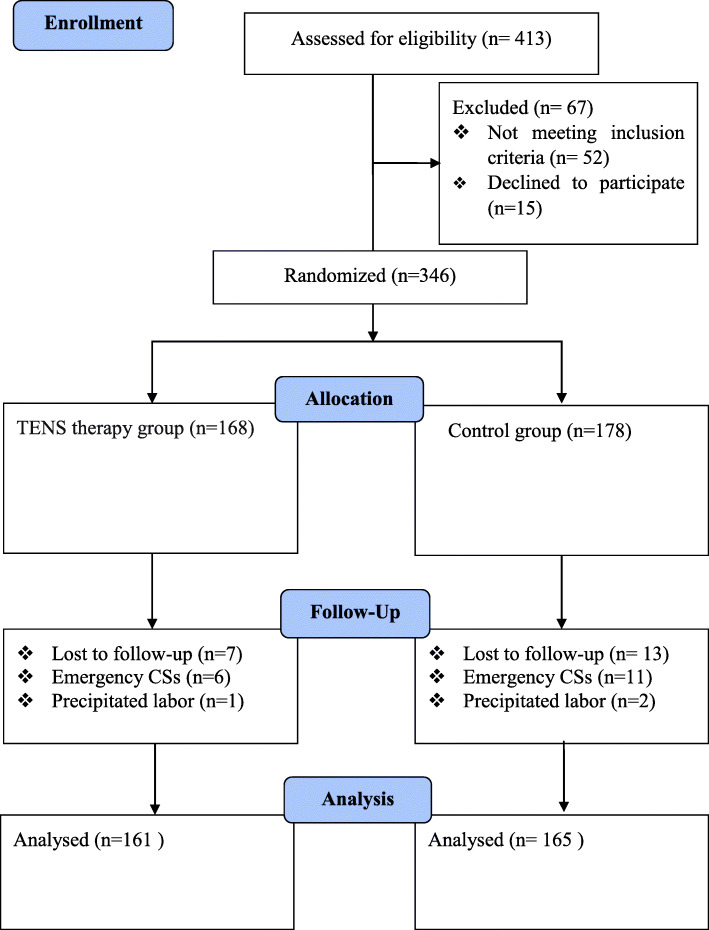
Consolidated Standards of Reporting Trials (CONSORT) flow diagram describing participant allocation in this study

We recruited a total number of 413 pregnant women, 346 of whom were eligible for the study, but 20 were excluded due to emergency cesarean sections and precipitated labor. Thus, the researcher randomly assigned 326 women into an experimental group (*n* = 161) and a control group (*n* = 165) Fig. 2.

Table 1 presents the baseline characteristics of the participants in each group

**Table 1:** Participant Characteristics

Characteristics	Experimental group (***N*** = 161)	Control group (***N*** = 165)	t-value	***p***- value
**Age in years**	29.32 ± 3.44	28.61 ± 3.58	−1.83	0.069
**Body mass index (Kg/m**^**2**^**)**	25.36 ± 2.76	25.29 ± 2.61	−0.25	0.800
**Gravidity**	1.69 ± 1.00	1.87 ± 0.93	1.72	0.087
**Parity**	1.26 ± 0.60	1.30 ± 0.46	0.61	0.540
**Gestation age in weeks**	39.13 ± 0.80	39.08 ± 0.98	−0.52	0.602
**Cervical dilatation in centimeters**	3.98 ± 0.16	4.00 ± 0.19	1.28	0.200

There was no statistically significant difference between the groups in terms of maternal age, body mass index, gravidity, parity, gestation age, and cervical dilatation.

There was no statistically significant difference in mean VAS scores before intervention between the groups (*p* > 0.05, Table 2). However, the experimental group had a statistically significantly lower mean VAS scores than the control group at 30, 60, and 120 min post-intervention and 2–24 h post-delivery (*p* < 0.001).

**Table 2:** Comparison of mean VAS scores in two groups

Pain Score	Experimental group (***N*** = 161)	Control group (***N*** = 16)	t-value	***p***-value
**Immediately after the randomization**	5.56 ± 1.56	5.64 ± 1.66	0.43	0.665
**30 min after TENS therapy**	5.67 ± 1.71	7.40 ± 1.40	9.96	0.001
**60 min after TENS therapy**	5.89 ± 1.92	8.78 ± 1.32	15.81	0.001
**120 min after TENS therapy**	5.45 ± 1.74	9.29 ± 1.39	22.05	0.001
**2–24 h after delivery**	6.02 ± 1.53	9.43 ± 0.98	23.86	0.001

The experimental group demonstrated a statistically significant shorter duration of the active labor phase than the control group (*p* < 0.001). Nevertheless, there was no statistically significant difference observed regarding the time of the second and third stages of labor (*p* > 0.05, Table 3).

**Table 3:** Comparison of duration of labor in two group

Duration of labor	Experimental group (***N*** = 161)	Control group (***N*** = 165)	t-value	***p***-value
**Duration of active phase in minutes**	172.25 ± 81.09	272.15 ± 110.41	9.32	0.001
**Duration of second stage in minutes**	38.45 ± 28.21	42.53 ± 29.61	−1.27	0.204
**Duration of third stage in minutes**	7.84 ± 7.81	8.61 ± 16.64	0.53	0.596

There was no statistically significant difference between the groups in terms of the first and fifth Apgar score, oxytocin usage, and postpartum hemorrhage between the two groups (*p* > 0.05, Table 4)

**Table 4:** Comparison of Apgar scores, blood loss, and oxytocin usage in two groups

Characteristics		The experimental group (***N*** = 161)	Control group (***N*** = 165)	Test statistic	***p***-value
**Apgar score (mean ± SD)**	At 1st minute	8.91 ± 0.42	8.91 ± 0.38	− 0.15 ^a^	0.88
	At 5th minutes	9.96 ± 0.23	9.97 ± 0.17	− 0.58 ^a^	0.56
**Oxytocin use**	No	127 (78.88%)	136 (82.42%)	0.33 ^b^	0.3
	Yes	34 (21.12%)	29 (17.58%)	0.01^a^	0.92
**Blood loss during delivery in** ***ml*** ^***C***^ **(mean ± SD)**		212.42 ± 58.64	211.82 ± 52.06		
**Baby wight in g** ^**d**^		3247 ± 371.63	3321.01 ± 406.18	−1.59 ^a^	0.11

^a^ Independent samples t-test; ^b^ Chi-square test; ^c^ Millilitres; ^d^ Grams

## Discussion

According to this study, there was no statistically significant difference in mean VAS scores before intervention between participants in the experimental and control group. However, the experimental group had a statistically significantly lower mean VAS scores than the control group at 30, 60, and 120 min after TENS therapy. These results were consistent with the previous studies [25–30]. However, two systematic reviews reported little difference in pain ratings between TENS and control groups [17, 22]. Van der Spank et al. revealed that the TENS group’s pain score was lower than in the control group. Still, there was no statistically significant difference in demand for epidural analgesia between the two groups [31]. According to two meta-analyses, there was no statistically significant difference in pain scores between the TENS and placebo groups [23, 24]. Previous trials used traditional TENS units, but we used high technology Bio-feedback TENS system.

Our study showed that the experimental group had a statistically significant shorter duration of the active labor phase than the control group. These results are consistent with other studies [26–28]. The utilization of both high and low-frequency TENS system parameters explains this finding. These parameters increase β-endorphins and methionine-enkephalin concentration and the production of inhibitory neurotransmitters such as GABA (gamma-aminobutyric acid) and serotonin but exhibiting neurotransmitters release (aspartate and glutamate) reduced. These natural analgesics substances inhibit the production of catecholamines [19, 20]. According to our study findings, the TENS therapy use during labor did not affect the childbirth process’s consequences, the maternal outcomes, and the fetal outcomes. The researchers recorded no advanced effects among women in the experimental group. These results are similar to other studies [26–32].

Participants in the experimental group had lower pain scores than the control group, 2–24 h post-delivery. The women’s request for analgesia adjustments to a level below the pain threshold during varying intensity contractions illustrates this concept. Thus, gaining control over the uterine contractions and treatment intensity. Having an influence on one’s care and feeling a sense of control are essential factors in labor pain management [33, 34]. Also, the TENS system mechanism’s deactivation of limbic areas could have reduced the pain’s emotional aspects, such as fear and anxiety.

Nevertheless, our study has some limitations. First, a double-blind study was difficult to conduct due to the nature of the intervention. Second, this was a small sample size and single-centered analysis based on one geographical area; thus, results cannot be generalized to the whole population. Future large sample size trials are necessary to verify the use of TENS. Third, we used VAS to quantify participants’ labor pain intensity, which is very subjective. Last, this study dealt with low-risk pregnant women and excluded those with high-risk pregnancy and previous history of using TENS.

## Conclusions

Our results indicate that the TENS therapy can be used as a non-pharmacologic therapy to reduce labor pain and shorten the active phase duration. Besides, the treatment seems to be safe for both mother and the fetus.

## Data Availability

The datasets analyzed during this study are available from the corresponding author on reasonable request.

## Abbreviations

TENS: Transcutaneous electrical nerve stimulation
VAS: Visual analog scale
CONSORT: Consolidated Standards of Reporting Trials
